# Decentralized Cooperative Localization with Fault Detection and Isolation in Robot Teams

**DOI:** 10.3390/s18103360

**Published:** 2018-10-08

**Authors:** Mei Wu, Hongbin Ma, Xinghong Zhang

**Affiliations:** 1 State Key Laboratory of Intelligent Control and Decision of Complex Systems, Beijing Institute of Technology, Beijing 100081, China; 3120150402@bit.edu.cn; 2 Department of Automatic Control, Henan Institute of Technology, Xinxiang 453000, China; hongxing0603@163.com

**Keywords:** robot localization, mobile robot, distributed cooperative localization, fault detection and isolation

## Abstract

Robot localization, particularly multirobot localization, is an important task for multirobot teams. In this paper, a decentralized cooperative localization (DCL) algorithm with fault detection and isolation is proposed to estimate the positions of robots in mobile robot teams. To calculate the interestimate correlations in a distributed manner, the split covariance intersection filter (SCIF) is applied in the algorithm. Based on the split covariance intersection filter cooperative localization (SCIFCL) algorithm, we adopt fault detection and isolation (FDI) to improve the robustness and accuracy of the DCL results. In the proposed algorithm, the signature matrix of the original FDI algorithm is modified for application to DCL. A simulation-based comparative study is conducted to demonstrate the effectiveness of the proposed algorithm.

## 1. Introduction

Robots have become increasingly prevalent in many domains, such as medical services and security [1]. Tasks assigned to robots such as rescue [2] are becoming increasingly complex for a single robot to address, making multirobot cooperation imperative. The cooperation intrinsic to a multirobot team makes it possible to execute tasks such as rescue which may be difficult or difficult with a single, highly capable robot; moreover, the presence of more than one robot makes the team fault-tolerant. The net effect is that a robot team can reliably execute its task even when its working environment changes.

Accurate position information is very important for cooperation in a multirobot team. For a single robot, the localization process is performed based only on its own sensor data, such as Global Positioning System (GPS) data [3], camera data [4], or laser scanner data [5,6]. In a multirobot team, however, sensor data collected from inter-robot observations can be considered in the process of cooperative multirobot localization. It is believed that cooperative localization (CL) methods using sensor data from inter-robot observations will outperform traditional single-robot localization methods [7].

**Figure 1:**
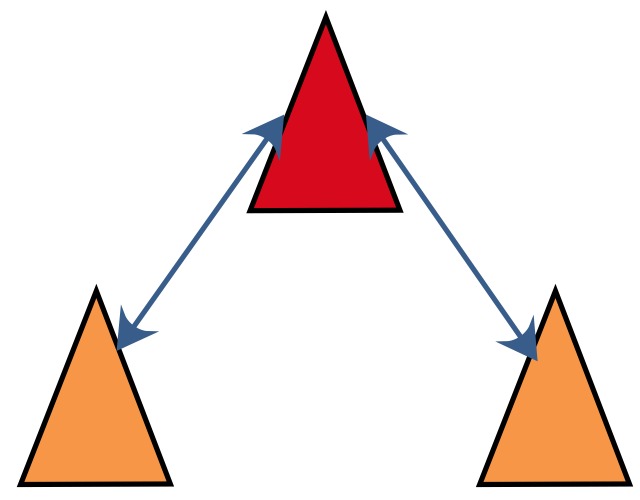
Communication graph for CCL.

The idea of CL was first proposed in [8], in which the robot team was considered to be divided into two groups. Each group alternately remained stationary to serve as a landmark for the other group. In [9], the premise of the stationarity of one group of robots is canceled. To date, many CL algorithms have been proposed. Among these algorithms, the simplest is centralized cooperative localization (CCL) [10]. In CCL, all information, including observation information and position information from the whole team, is transmitted to a fusion center, which maintains only one global state, as shown in Figure 1.

However, CCL has clear disadvantages. First, the communication and computational costs of CCL are very high since all computational loads are concentrated at the fusion center. Second, if a fault occurs in the fusion center, then the whole CCL system will be corrupted. Finally, given the finite communication and computational capabilities of a practical system, it is difficult to continue to use only one fusion center to achieve CL for the entire team as the size of the team increases.

Decentralized cooperative localization (DCL) has proven to be a desirable solution [11] to resolve these problems, because both the computational and communication burdens placed on each robot in the team are lower. In DCL, each robot handles only its own local information. Moreover, in this paper, to calculate the interestimate correlations in a distributed manner, the split covariance intersection filter (SCIF) [12] is applied. In this algorithm, two covariance states, namely, independent covariance and dependent covariance, are maintained in each robot.

To improve the robustness of the robot team, fault detection and isolation (FDI) [13,14,15] are needed. In [16], an FDI algorithm was applied in CCL. However, the FDI algorithms cited above are designed for CCL and are not applicable in DCL due to the limited sensor information available. To improve the performance of fault detection, a decision algorithm that maximizes the amount of information transformation is adopted, as introduced in Section 3. To enable the application of FDI in DCL, the isolation rule is modified. The signature matrix is simplified by combining certain fault types, and a new Kullback–Leibler (KL) residual is added.

The remainder of this paper is organized as follows. The theoretical background of the DCL and FDI algorithm is introduced in Section 2. We describe the concepts of DCL with the SCIF and FDI and introduce the corresponding algorithms in Section 3. The experiments and results are introduced in Section 4. The last section concludes the paper.

## 2. Background

In this section, some background knowledge is introduced.

Four assumptions are made in this paper. First, each robot is assumed to be equipped with one motion sensor. The equipped sensors can report the motion data on of the robots. No single-robot localization method based on other sensors is considered in these simulations.

Second, the robots are assumed to be able to capture the relative poses of other robots within their range of perception. A robot that is perceived is called a neighboring robot in this paper. Each robot can share its relative measurements and motion states with its neighboring robots via inter-robot communication.

**Figure 2:**
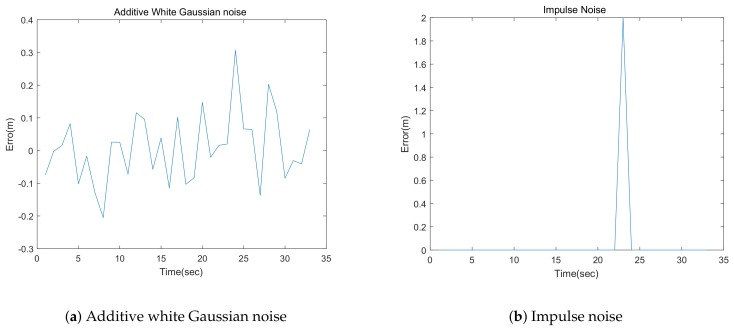
Additive white Gaussian noise and impulse noise.

Third, two types of noises are considered in the simulations: additive white Gaussian noise and impulse noise. Here, additive white Gaussian noise consists of unbiased normally distributed errors with a fixed standard deviation, as shown in Figure 2a, whereas impulse noise consists of unpredictable errors that simulate certain unexpected situations, such as poor odometric measurements and noisy and distorted measurements, as shown in Figure 2b.

**Figure 3:**
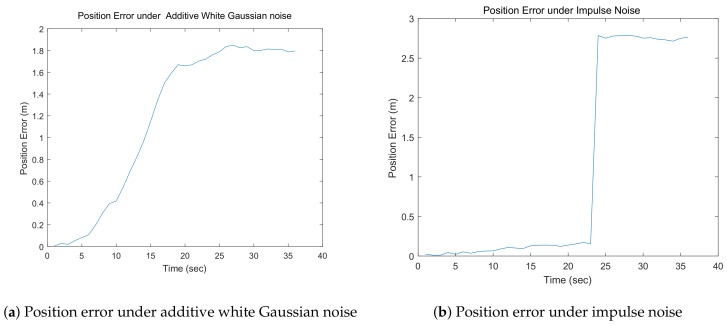
Position errors under additive white Gaussian noise and impulse noise.

To demonstrate the features of these two types of noise in our simulation, the localization errors of a robot under these two types of noise without filtering are shown in Figure 3a,b. As shown in these figures, the localization error of the robot increases smoothly under additive white Gaussian noise, whereas it increases suddenly under impulse noise.

Fourth, the members of the robot team are assumed to be subject to different levels of positioning error. When one robot changes its orientation to avoid an obstacle, the accuracy of its motion model will be lower than those of its neighboring robots because the amplitude of the noise is related to the yaw rate and the yaw rate of this robot will be larger than those of the other robots on the team.

### 2.1. Split Covariance Intersection Filter

As mentioned above, in DCL with the SCIF, each robot executes a localization algorithm to calculate its own position. This subsection provides a brief introduction to the concept of the SCIF, which enables the fusion of two different estimates with an unknown correlation.

Given two estimates $\left\{X_{1},P_{1}\right\}$ and $\left\{X_{2},P_{2}\right\}$, where *X* denotes the estimated state vector and *P* denotes the estimated covariance, the following fused estimate {X,P} can be calculated using the Kalman filter:

(1) $$\begin{array}{cc} P^{-1}= & P_{1}^{-1}+P_{2}^{-1},\\ X= & P(P_{1}^{-1}X_{1}+P_{2}^{-1}X_{2}). \end{array}$$

However, one premise of the Kalman filter that may not be satisfied is that the estimate and the observation are independent of each other. In other words, the estimate and the observation are correlated, Equation (1) might yield inconsistent results, and the fused result might be overconfident.

One widely used tool for handling the fusion of estimates with unknown correlations is the covariance intersection filter (CIF) [17]. In the CIF approach, a parameter *w* is added to the fusion formulae:

(2) $$\begin{array}{cc} P^{-1}= & {\left(\frac{P_{1}}{w}\right)}^{-1}+{\left(\frac{P_{2}}{1-w}\right)}^{-1},\\ X= & P({\left(\frac{P_{1}}{w}\right)}^{-1}X_{1}+{\left(\frac{P_{2}}{1-w}\right)}^{-1}X_{2}), \end{array}$$

where *w*, which takes values in the interval [0, 1], is chosen to minimize *P*.

Although the CIF alleviates the overconfidence problem, it also has clear drawbacks, mainly because it treats the estimates to be fused as completely interdependent values. Thus, the fused result estimated with the CIF is pessimistic. To further mitigate this problem, the SCIF [12] has been introduced. In the algorithm of SCIF, the estimates to be fused are split into two parts: an independent part and a dependent part. Thus, the estimate $\left\{X_{1},P_{1}\right\}$ and the observation $\left\{X_{2},P_{2}\right\}$ to be fused are reformulated as $\left\{X_{1},P_{1d}+P_{1i}\right\}$ and $\left\{X_{2},P_{2d}+P_{2i}\right\}$, respectively, where $P_{1d}$ corresponds to the dependent part of $X_{1}$ and $P_{1i}$ corresponds to the dependent part of $X_{1}$. The formula of SCIF can be written as

(3) $$\begin{array}{cc} P_{1}= & \frac{P_{1d}}{w}+P_{1i},\\ P_{2}= & \frac{P_{2d}}{1-w}+P_{2i},\\ P^{-1}= & P_{1}^{-1}+P_{2}^{-1},\\ X= & P(P_{1}^{-1}X_{1}+P_{2}^{-1}X_{2}),\\ P_{i}= & P(P_{1}^{-1}P_{1i}P_{1}^{-1}+P_{2}^{-1}P_{2i}P_{2}^{-1})P,\\ P_{d}= & P-P_{i}, \end{array}$$

where *w*, which takes values in the interval [0, 1], is chosen to minimize *P*.

### 2.2. Statistical Decision Theory Based on an Information Theoretic Approach

In this subsection, statistical decision theory based on an information theoretic approach is introduced. In this algorithm, the decision is evaluated based on the mutual information $I(S,S_{a})$. The decision rule δ(x), which is based on an observation *x*, can be written as shown in Equation (4).

(4) $$\delta \left(x\right)=\underset{S_{a}}{\arg\max}I(S,S_{a})$$

Here, the decision $S_{a}$ is chosen to maximize $I(S,S_{a})$. The input state *x* represents the observation of the fault state of the detection system, which is conditioned on the true state, represented by *S*.

The state space of *S* consists of two values: $s_{0}$ or $s_{1}$, where $s_{0}$ represents the absence of fault and $s_{1}$ represents the presence of fault. Similarly the decision result $S_{a}$ may only take one of these two values.

The value of $I(S,S_{a})$ can be calculated using Equation (5):

(5) $$I\left(S,S_{a}\right)=\sum_{S}\sum_{S_{a}}p(S,S_{a})\log\left(\frac{p(S|S_{a})}{p(S_{a})}\right),$$

where the various probabilities that appear in this expression are defined as follows:

$p(S|S_{a})$:

(6) $$p\left(S|S_{a}\right)=\left\{\begin{array}{cc} p(S=s_{0}|S_{a} & =s_{0})=1-P_{F},\\ p(S=s_{0}|S_{a} & =s_{1})=P_{F},\\ p(S=s_{1}|S_{a} & =s_{0})=1-P_{D},\\ p(S=s_{1}|S_{a} & =s_{1})=P_{D}. \end{array}\right.$$

$p(S_{a})$:

(7) $$\begin{array}{cc} p(S_{a}=s_{0}) & =P_{0}\left(1-P_{F}\right)+\left(1-P_{0}\right)\left(1-P_{D}\right),\\ p(S_{a}=s_{1}) & =P_{0}P_{F}+\left(1-P_{0}\right)P_{D}. \end{array}$$

$p(S,S_{a})$:

(8) $$\begin{array}{c} p\left(S,S_{a}\right)\left\{\begin{array}{cc} p(S=s_{0},S_{a} & =s_{0})=\left(1-P_{F}\right)P_{0},\\ p(S=s_{0},S_{a} & =s_{1})=P_{F}P_{0},\\ p(S=s_{1},S_{a} & =s_{0})=\left(1-P_{D}\right)\left(1-P_{0}\right),\\ p(S=s_{1},S_{a} & =s_{1})=P_{D}\left(1-P_{0}\right). \end{array}\right. \end{array}$$

Here, $P_{D}$ is the probability of correctly concluding that there are faulty sensors when faulty sensors are present in the robot system, $P_{F}$ is the probability of incorrectly concluding that there are faulty sensors when no faulty sensors are present in the robot system, and $P_{0}$ is a priori probability of the absence of faulty sensors in the robot system, whose initial value is known.

The values of $P_{D}$ and $P_{F}$ are calculated as shown in Equation (9):

(9) $$\begin{array}{c} P_{D}=\int_{\lambda }^{\infty }f\left(x|S=s_{1}\right)dx,\\ P_{F}=\int_{\lambda }^{\infty }f\left(x|S=s_{0}\right)dx. \end{array}$$

Additionally, the probability density functions $f(x|S=s_{0})$ and $f(x|S=s_{1})$ are computed from the data distributions of *x* in the faulty and nonfaulty cases, respectively.

By substituting Equations (6)–(8) into Equation (5), we can obtain Equation (10):

(10) $$\begin{array}{cc} I(S,S_{a})= & P_{0}\left(1-P_{F}\right)\log\left(\frac{1-P_{F}}{P_{0}\left(1-P_{F}\right)+\left(1-P_{0}\right)\left(1-P_{D}\right)}\right)\\ & +P_{0}P_{F}\log\left(\frac{P_{F}}{P_{0}P_{F}+\left(1-P_{0}\right)P_{D}}\right)\\ & +\left(1-P_{0}\right)\left(1-P_{D}\right)\log\left(\frac{1-P_{D}}{P_{0}\left(1-P_{F}\right)+\left(1-P_{0}\right)\left(1-P_{D}\right)}\right)\\ & +\left(1-P_{0}\right)\left(P_{D}\right)\log\left(\frac{P_{D}}{P_{0}P_{F}+\left(1-P_{0}\right)P_{D}}\right). \end{array}$$

The threshold *t* of the likelihood ration that maximizes $I(S,S_{a})$ is determined as follows. First, the derivative of $I(S,S_{a})$ with respect to $P_{F}$ is calculated:

(11) $$\begin{array}{cc} \partial I/\partial P_{F} & =-P_{0}\log\frac{1-P_{F}}{P_{F}}+\left[P_{0}+t\left(1-P_{0}\right)\right]\log\left(p(S_{a}=s_{0})\right)\\ & -t\left(1-P_{0}\right)\log\frac{1-P_{D}}{P_{D}}\\ & -\left[P_{0}+t\left(1-P_{0}\right)\right]\log\left(p(S_{a}=s_{1})\right). \end{array}$$

Set this derivative is equal to zero. We obtain that

(12) $$\begin{array}{c} t=\frac{-P_{0}\left[\log\left(p\left(S_{a}=s_{0}\right)/p\left(S_{a}=s_{1}\right)\right)-\log\left(\left(1-P_{F}\right)/P_{F}\right)\right]}{\left(1-P_{0}\right)\left[\log\left(p\left(S_{a}=s_{0}\right)/p\left(S_{a}=s_{1}\right)\right)-\log\left(\left(1-P_{D}\right)/P_{D}\right)\right]}. \end{array}$$

Here,

(13) $$\begin{array}{c} t=\frac{\partial P_{D}}{\partial P_{F}}. \end{array}$$

With this threshold *t*, the decision rule δ() can be written as

(14) $$\delta \left(x\right)=\left\{\begin{array}{cc} s_{1} & l(x)\geq t,\\ s_{0} & l(x)<t. \end{array}\right.$$

Here, l(x) is a likelihood ratio that is calculated as follows:

(15) $$\begin{array}{c} l\left(x\right)=\frac{f_{x}\left(x|s_{1}\right)}{f_{x}\left(x|s_{0}\right)}. \end{array}$$

Likely wise Equation (14) can be expressed as

(16) $$\delta \left(x\right)=\left\{\begin{array}{cc} s_{1} & x\geq \lambda ,\\ s_{0} & x<\lambda . \end{array}\right.$$

Here λ is calculated by algorithm where is chosen from the a set λ consisting of values between $\lambda_{max}$ and $\lambda_{min}$ (shown in Algorithm 1).

**Algorithm 1** Algorithm of the thresholding calculation:
1:
i←1
2:
$I(S,S_{a})\leftarrow 0$
3:**while** i<max **do**
4: calculate $P_{Fi}$ and $P_{Di}$ with $\lambda_{i}$ by Equation (9)5: calculate $I{\left(S,S_{a}\right)}_{i}$ with $P_{Fi}$ and $P_{Di}$ by Equation (10)6: **if** $I{\left(S,S_{a}\right)}_{i}>I\left(S,S_{a}\right)$ **then**7:  $I\left(S,S_{a}\right)\leftarrow I{\left(S,S_{a}\right)}_{i}$
8:  $\lambda \leftarrow \lambda_{i}$
9: **end if**10: i←i+1
11:**end while**


## 3. Materials and Methods

**Figure 4:**
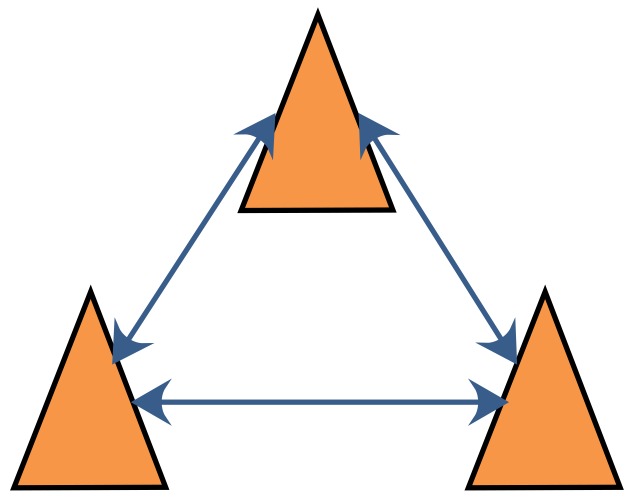
Communication graph for DCL.

The DCL algorithm with FDI is introduced in this section. The communication graph of DCL in a robot team with three robot members, where each robot communicates only with its neighbors, is shown in Figure 4. As shown in this figure, there is no fusion center in DCL; instead, every robot executes its own localization and FDI algorithm to calculate its own position. In contrast with the fusion center in CCL, which can obtain all the information that it needs, in DCL, every robot can obtain information only from its neighbors. For this reason, the information available to every robot in DCL is limited, and the FDI algorithms designed for CCL are not applicable to DCL. To enable the application of FDI in DCL, a novel FDI algorithm is introduced in this section.

### 3.1. Decentralized Cooperative Localization Algorithm

In this section, the method for achieving DCL using the SCIF (abbreviated henceforth as SCIFCL) is introduced. The SCIFCL algorithm is presented as Algorithm 2.

**Algorithm 2** DCL Algorithm with the SCIF (SCIFCL):
1:**for** i=1 to *N* **do**2: $X_{k/k-1}^{i}=f\left(X_{k-1}^{i},u_{k-1}^{i}\right)$
3: $P_{k/k-1}^{i}=F_{k-1}^{i}P_{k-1}^{i}{F^{i}}_{k-1}^{T}+G_{k-1}^{i}{\left(Q_{u}\right)}_{k-1}^{i}{G^{i}}_{k-1}^{T}$
4: $P_{k/k-1}^{i\_in}=F_{k-1}^{i}P_{k-1}^{i\_in}{F^{i}}_{k-1}^{T}+G_{k-1}^{i}{\left(Q_{u}\right)}_{k-1}^{i}{G^{i}}_{k-1}^{T}$
5: $P_{k/k-1}^{i\_d}=P_{k/k-1}^{i}-P_{k/k-1}^{i\_in}$
6: **if** there exists a relative measurement $Z_{k}^{ji}$ between robot *j* and robot *i* **then**7:  $X_{k/k-1}^{i*}=h\left(X_{k/k-1}^{j},Z_{k}^{ji}\right)$
8:  $P_{k/k}^{i}=\left(1-K\right)\left(P_{k/k-1}^{i\_d}/w+P_{k/k-1}^{i\_in}\right)$
9:  $X_{k/k}^{i}=X_{k/k-1}^{i}+K\left(X_{k/k-1}^{i*}-X_{k/k-1}^{i}\right)$10: **end if**11:**end for**


The state model of robot *i* in a multiple mobile robots team can be expressed as

(17) $$\begin{array}{cc} X_{k/k-1}^{i}= & f(X_{k-1}^{i},u_{k-1}^{i})\\ = & \left(\begin{array}{c} x_{k-1}^{i}\\ y_{k-1}^{i}\\ \theta_{k-1}^{i} \end{array}\right)+\left(\begin{array}{c} \Delta_{k-1}^{i}cos\left(\theta_{k-1}^{i}+\frac{v_{k-1}^{i}}{2}\right)\\ \Delta_{k-1}^{i}sin\left(\theta_{k-1}^{i}+\frac{v_{k-1}^{i}}{2}\right)\\ v_{k-1}^{i} \end{array}\right). \end{array}$$

where $X_{k}^{i}$ is the state vector of robot *i*:

$$X_{k}^{i}={\left[x_{k}^{i},y_{k}^{i},\theta_{k}^{i}\right]}^{T},$$

and

$$X_{k-1}={\left[X_{k-1}^{1},X_{k-1}^{2},...,X_{k-1}^{i},...\right]}^{T}.$$

Here, $\Delta_{k-1}^{i}$ and $v_{k-1}^{i}$ are the elementary displacement and rotation, respectively, of robot *i*.

In summary, the state model of the multirobot system consists of differentially driven robots governed by a linear control law.

And the Jacobian matrices $F_{k-1}^{i}=\frac{\partial f}{\partial X}$ and $G_{k-1}^{i}=\frac{\partial f}{\partial u}$ are written as

(18) $$F_{k-1}^{i}=\left(\begin{array}{ccc} 1 & 0 & -\Delta_{k-1}^{i}sin\left(\theta_{k-1}^{i}+\frac{v_{k-1}^{i}}{2}\right)\\ 0 & 1 & \Delta_{k-1}^{i}cos\left(\theta_{k-1}^{i}+\frac{v_{k-1}^{i}}{2}\right)\\ 0 & 0 & 1 \end{array}\right)$$

and

(19) $$G_{k-1}^{i}=\left(\begin{array}{cc} cos(\theta_{k-1}^{i}+\frac{v_{k-1}^{i}}{2}) & -\frac{1}{2}\Delta_{k-1}^{i}sin\left(\theta_{k-1}^{i}+\frac{v_{k-1}^{i}}{2}\right)\\ sin(\theta_{k-1}^{i}+\frac{v_{k-1}^{i}}{2}) & \frac{1}{2}\Delta_{k-1}^{i}cos\left(\theta_{k-1}^{i}+\frac{v_{k-1}^{i}}{2}\right)\\ 0 & 1 \end{array}\right).$$

In DCL, in addition to the covariance of the robot itself, $P_{k/k-1}^{i}$, two additional covariances, $P_{k/k-1}^{i\_in}$ and $P_{k/k-1}^{i\_d}$, are also calculated as follows:

(20) $$\begin{array}{cc} P_{k/k-1}^{i}= & F_{k-1}^{i}P_{k-1}^{i}{F^{i}}_{k-1}^{T}+G_{k-1}^{i}{\left(Q_{u}\right)}_{k-1}^{i}{G^{i}}_{k-1}^{T},\\ P_{k/k-1}^{i\_in}= & F_{k-1}^{i}P_{k-1}^{i\_in}{F^{i}}_{k-1}^{T}+G_{k-1}^{i}{\left(Q_{u}\right)}_{k-1}^{i}{G^{i}}_{k-1}^{T},\\ P_{k/k-1}^{i\_d}= & P_{k/k-1}^{i}-P_{k/k-1}^{i\_in}. \end{array}$$

When robot *i* is observed by robot *j*, the measurement model can be written as

Thus, the measurement of $X_{k}^{i}$ can be written as

(21) $$\begin{array}{cc} X_{k/k-1}^{i*}= & h(X_{k/k-1}^{j},Z_{k}^{ji})\\ = & \left(\begin{array}{c} x_{k/k-1}^{j}+x_{k}^{ji}cos\theta_{k/k-1}^{j}-y_{k}^{ji}sin\theta_{k/k-1}^{j}\\ y_{k/k-1}^{j}+x_{k}^{ji}sin\theta_{k/k-1}^{j}+y_{k}^{ji}cos\theta_{k/k-1}^{j}\\ \theta_{k/k-1}^{j}+\theta_{k}^{ji} \end{array}\right), \end{array}$$

where

$$Z_{k}^{ji}={\left[x_{k}^{ji},y_{k}^{ji},\theta_{k}^{ji}\right]}^{T}.$$

Then, the measurement and the observation to be fused are $\left\{X_{k/k-1}^{i},P_{k/k-1}^{i\_d}+P_{k/k-1}^{i\_in}\right\}$ and $\left\{X_{k/k-1}^{i*},P_{k/k-1}^{i*\_d}+P_{k/k-1}^{i*\_in}\right\}$, respectively, where

(22) $$\begin{array}{c} P_{k/k-1}^{i*}=C_{k}^{i*}P_{k/k-1}^{j}{\left(C_{k}^{i*}\right)}^{T}+D_{k}^{i*}{\left(D_{k}^{i*}\right)}^{T},\\ P_{k/k-1}^{i*\_in}=C_{k}^{i*}P_{k/k-1}^{j\_in}{\left(C_{k}^{i*}\right)}^{T}+D_{k}^{i*}{\left(D_{k}^{i*}\right)}^{T}. \end{array}$$

Here, the Jacobian matrices $C_{k}^{i*}=\frac{\partial X_{k/k-1}^{i*}}{\partial X_{k/k-1}^{j}}$ and $D_{k}^{i*}=\frac{\partial X_{k/k-1}^{i*}}{\partial Z_{k}^{ji}}$ can be expressed as

(23) $$\begin{array}{cc} C_{k}^{i*}= & \frac{\partial X_{k/k-1}^{i*}}{\partial X_{k/k-1}^{j}}\\ = & \left(\begin{array}{ccc} 1 & 0 & -x_{k}^{ji}sin\left(\theta_{k/k-1}^{j}\right)-y_{k}^{ji}cos\left(\theta_{k/k-1}^{j}\right)\\ 0 & 1 & x_{k}^{ji}cos\left(\theta_{k/k-1}^{j}\right)-y_{k}^{ji}sin\left(\theta_{k/k-1}^{j}\right)\\ 0 & 0 & 1 \end{array}\right),\\ D_{k}^{i*}= & \frac{\partial X_{k/k-1}^{i*}}{\partial Z_{k}^{ji}}. \end{array}$$

Applying the SCIF to the cooperative localization problem, we obtain that

(24) $$\begin{array}{c} {\left(P_{k/k}^{i}\right)}^{-1}={\left(\frac{P_{k/k-1}^{i\_d}}{w}+P_{k/k-1}^{i\_in}\right)}^{-1}+{\left(\frac{P_{k/k-1}^{i*\_d}}{1-w}+P_{k/k-1}^{i*\_in}\right)}^{-1}\\ X_{k/k}^{i}=P_{k/k}^{i}\left[{\left(\frac{P_{k/k-1}^{i\_d}}{w}+P_{k/k-1}^{i\_in}\right)}^{-1}X_{k/k-1}^{i}+{\left(\frac{P_{k/k-1}^{i*\_d}}{1-w}+P_{k/k-1}^{i*\_in}\right)}^{-1}X_{k/k-1}^{i*}\right]. \end{array}$$

Writing the above equation in Kalman filter form, we obtain that

(25) $$\begin{array}{c} P_{k/k}^{i}=\left(1-K\right)\left(\frac{P_{k/k-1}^{i\_d}}{w}+P_{k/k-1}^{i\_in}\right) \end{array}$$

(26) $$\begin{array}{c} X_{k/k}^{i}=X_{k/k-1}^{i}+K\left(X_{k/k-1}^{i*}-X_{k/k-1}^{i}\right), \end{array}$$

where $K=\frac{\left(\frac{P_{k/k-1}^{i\_d}}{w}+P_{k/k-1}^{i\_in}\right)}{\left(\frac{P_{k/k-1}^{i\_d}}{w}+P_{k/k-1}^{i\_in}\right)+\left(\frac{P_{k/k-1}^{i*\_d}}{1-w}+P_{k/k-1}^{i*\_in}\right)}$.

And

(27) $$\begin{array}{cc} P_{k/k}^{i\_in}= & \left(1-K\right)P_{k/k-1}^{i\_in}{\left(1-K\right)}^{T}+KP_{k/k-1}^{i*\_in}K^{T}\\ P_{k/k}^{i\_d}= & P_{k/k}^{i}-P_{k/k}^{i\_in}. \end{array}$$

Thus, the fused result is $\left\{X_{k/k}^{i},P_{k/k}^{i\_d}+P_{k/k}^{i\_in}\right\}$.

### 3.2. Fault Detection and Isolation in Decentralized Cooperative Localization

After estimating the locations of the robot team via SCIFCL, the FDI algorithm is implemented. As mentioned above, in DCL, each member of the team communicates only with its neighbors, and thus, the information available to each robot for FDI is limited. To resolve this problem, an improved FDI algorithm is developed.

To evaluate the possibility of the presence of faulty sensors, the Kullback–Leibler divergence (KLD) between the results of estimation before and after the measurement update is calculated. The KLD is a measure of the extent of one probability distribution diverges from another. If f(x) and g(x) are two-dimensional Gaussian distributions, with respective means of $\mu_{1}$ and $\mu_{2}$ and covariance matrices $P_{1}$ and $P_{2}$, then the KLD can be calculated using Equation (28):

(28) $$KLD\left(f\left(x\right)||g\left(x\right)\right)=\frac{1}{2}\left[\mathrm{trace}\left({P_{2}}^{-1}P_{1}\right)+\log\left(\det\left(\frac{P_{2}}{P_{1}}\right)\right)-d+{\left(\mu_{1}-\mu_{2}\right)}^{T}P_{2}^{-1}\left(\mu_{1}-\mu_{2}\right)\right].$$

Here, the mean $X_{k/k-1}$ and the covariance matrix $P_{k/k-1}$ of the state estimate before the measurement update correspond to the prior probability distribution, while the mean $X_{k/k}$ and the covariance matrix $P_{k/k}$ of the state estimate after the measurement update correspond to the posterior probability distribution.

Since both the prior and posterior probability distributions are two-dimensional Gaussian distributions, each robot can use the information obtained from its neighbors to calculate $GKLD^{i}$, as an indicator of the possible presence of a fault of robot *i*, as shown in Equation (29).

(29) $$\begin{array}{cc} GKLD^{i}= & KLD(X_{k/k-1}^{i}||X_{k/k}^{i})\\ = & \frac{1}{2}[\mathrm{trace}\left({P_{k/k}^{i}}^{-1}P_{k/k-1}^{i}\right)+\log\left(\det\left(\frac{P_{k/k}^{i}}{P_{k/k-1}^{i}}\right)\right)-M\\ + & {\left(X_{k/k}^{i}-X_{k/k-1}^{i}\right)}^{T}{P_{k/k}^{i}}^{-1}\left(X_{k/k}^{i}-X_{k/k-1}^{i}\right)] \end{array}$$

With the threshold optimization algorithm mentioned in Section 2, the problem of fault detection in DCL is simplified to find the $S_{a}$ which maximizes the value of $\delta (GKLD^{i})$ in Equation (30).

(30) $$\delta \left(GKLD^{i}\right)=\underset{S_{a}}{\arg\max}I(S,S_{a})$$

Here the state space of *S* consists of two values: $s_{0}$ or $s_{1}$, where $s_{0}$ represents the absence of faulty sensors and $s_{1}$ represents the presence of faulty sensors.

And the threshold λ can be expressed as the function of $GKLD^{i}$.

(31) $$\delta \left(GKLD^{i}\right)=\left\{\begin{array}{cc} s_{1} & GKLD^{i}\geq \lambda \\ s_{0} & GKLD^{i}<\lambda \end{array}\right.$$

Here the threshold λ is obtained by Algorithm 1.

After fault detection, if a fault in a robot is detected, then the fault isolation procedure is applied to identify the faulty robot. KL residuals are calculated to isolate the fault. The number of KL residuals calculated depends on the number of inter-robot observations. For example, when robot *j* is observed by robot *i*, the KL residual $KL^{ij}$ is calculated as shown in Equation (32):

(32) $$\begin{array}{cc} KL^{ij}= & KLD(X_{k/k-1}^{i}||X_{k/k}^{ij})\\ = & \frac{1}{2}{\left[\mathrm{trace}\left({P_{k/k}^{ij}}^{-1}P_{k/k-1}^{i}\right)+\log\left(\det\left(\frac{P_{k/k}^{ij}}{P_{k/k-1}^{i}}\right)\right)-M+\left(X_{k/k}^{ij}-X_{k/k-1}^{i}\right)\right)}^{T}{P_{k/k}^{ij}}^{-1}\left(X_{k/k}^{ij}-X_{k/k-1}^{i}\right)]. \end{array}$$

Here, $X_{k/k}^{ij}$ denotes the estimation calculated by robot *i* based on its observation of robot *j*.

Each KL residual is sensitive to some faults and insensitive to others. For example, the value of $KL^{ij}$ depends on the odometers of robots *i* and *j* and the observation of robot *j* as measured by robot *i*.

(33) $$\begin{array}{cc} \delta (KL^{ij})= & \underset{S_{a}}{\arg\max}I(S,S_{a})\\ = & s_{1} \end{array}$$

If the value of $KL^{ij}$ satisfies Equation (33), then there may be a fault in the odometer of robot *j* or *i* or in robot *i*’s observation of robot *j*. The corresponding signal value is equal to 1. As an illustrative example, the signature matrix of robot 1 is given in Table 1.

Since the authors of [16] utilized lidar and Kinect to capture the relative position of other robot, the observation here may represent the sensor information from these two sensors.

However, it is difficult to isolate the faulty sensors with signature matrices described above. To enable FDI in DCL, the signature matrices are simplified by combining the information on the observation and the odometer sensor for each robot. Here, the grouped observation information for each robot is sensitive to faults in both the observation and the odometer sensor of that robot. The corresponding signal value will equal to 1 when either the observation or the odometer sensor is faulty. Thus, the signature matrices are modified as shown in Table 2, Table 3 and Table 4 for the case of a robot team with three members.

However, after this simplification, there is one fault type that cannot be isolated. If faults exist in both sensors, then the signature matrices will both be equal to (1, 1), which is same with the case that faults exist in both observations. Thus, no observation information will be accepted, even if these sensors are free of faults.

To solve this problem, we add another set of KLD entries to the signature matrix based on the measured divergence between the observations of the two robots being observed. Thus, the signature matrices are modified to those shown in Table 5, Table 6 and Table 7. The proposed signature matrices can be used to identify more fault types than is possible with the classic isolation algorithm.

(34) $$\begin{array}{cc} KL^{ik/ij}= & KLD(X_{k}^{i/k}||X_{k}^{i/j})\\ = & \frac{1}{2}{\left[\mathrm{trace}\left({P_{k}^{i/j}}^{-1}P_{k}^{i/k}\right)+\log\left(\det\left(\frac{P_{k}^{i/j}}{P_{k}^{i/k}}\right)\right)-M+\left(X_{k}^{i/j}-X_{k}^{i/k}\right)\right)}^{T}{P_{k}^{i/j}}^{-1}\left(X_{k}^{i/j}-X_{k}^{i/k}\right)] \end{array}$$

Here, $X_{k}^{i/k}$ denotes the observation of robot *k* as measured by robot *i*.

(35) $$\begin{array}{cc} \delta (KL^{ik/ij})= & \underset{S_{a}}{\arg\max}I(S,S_{a})\\ = & s_{1} \end{array}$$

If the value of $KL^{ik/ij}$ satisfies Equation (35), the corresponding signal value is equal to 1.

## 4. Results

**Figure 5:**
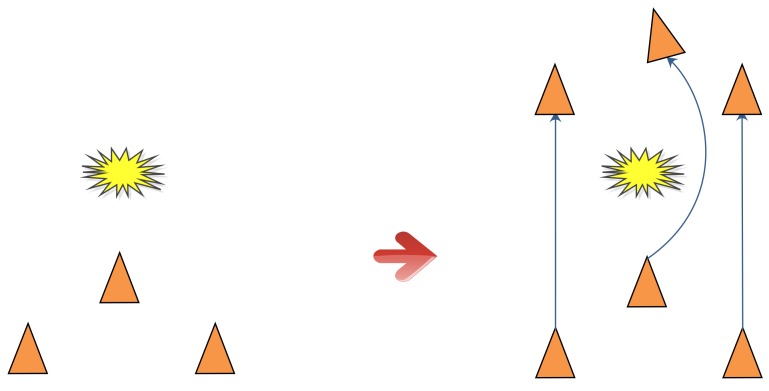
Simulation scenario: a team of three robots.

To validate the FDI algorithm proposed in this paper, four simulations are performed. The simulations reported in this paper were conducted in MATLAB. The background for the simulations was based on a real scenario in which a team of robots were moved forward in a certain formation while maneuvering to avoid an obstacle ahead. To maintain the formation of the team, one robot adjusted its orientation, as shown in Figure 5.

The localization results obtained for the robot team using the SCIFCL algorithm and the SCIFCL algorithm with FDI were tested in four simulations. In the first two simulations, only additive white Gaussian noise was considered, and the distance between neighboring robots was varied from 2 m to 1 m. The velocity of each robot was approximately 0.3 m/s. The additive white Gaussian noise added to the motion measurements introduced standard errors of 0.1 m/s in velocity and 0.005 rad/s in yaw rate. The absolute positioning measurement period was set to 1 s. The standard errors in relative positioning were set to 0.1 m in relative position and 0.005 rad in relative orientation. Time delay was not considered in these simulations.

The simulations consisted of two primary stages. In the first stage, one robot was randomly chosen as the leader, and each of the other robots used its relative measurements of the leader robot until its own state estimation converged. Then, in the second stage, the SCIFCL algorithm and the SCIFCL algorithm with FDI were executed simultaneously, and the robot localization errors associated with these two algorithms were recorded.

**Figure 6:**
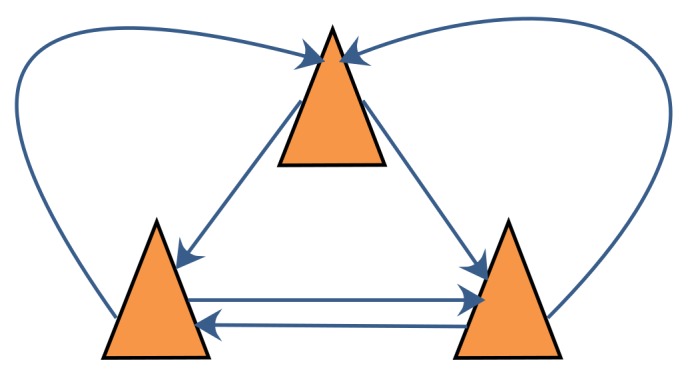
Communication graph for 3 robots.

**Figure 7:**
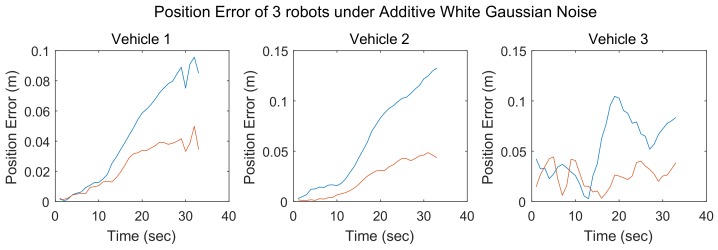
Position errors of 3 robots under additive white Gaussian noise.

In simulation 1, the number of robots in the robot team was set to 3. The communication graph for the robot team is shown in Figure 6. The results of simulation 1 are shown in Figure 7, where each subfigure presents the localization errors of one team member with different localization methods. Here, the vertical axis represents the position error, and the horizontal axis represents the time step. The localization errors of the different methods are represented by lines of different colors. The blue line denotes the robot localization error achieved with the SCIFCL algorithm, and the red line denotes the robot localization error achieved with the SCIFCL algorithm with FDI.

**Figure 8:**
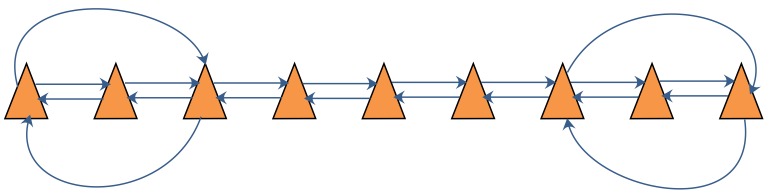
Communication graph for 9 robots.

In simulation 2, the number of robots in the robot team was set to be 9. Since at least two neighbor observations are needed for each robot to apply the SCIFCL algorithm with FDI, the communication graph for the robot team was defined as shown in Figure 8, with each robot using only the information obtained by observing the two nearest robots.

**Figure 9:**
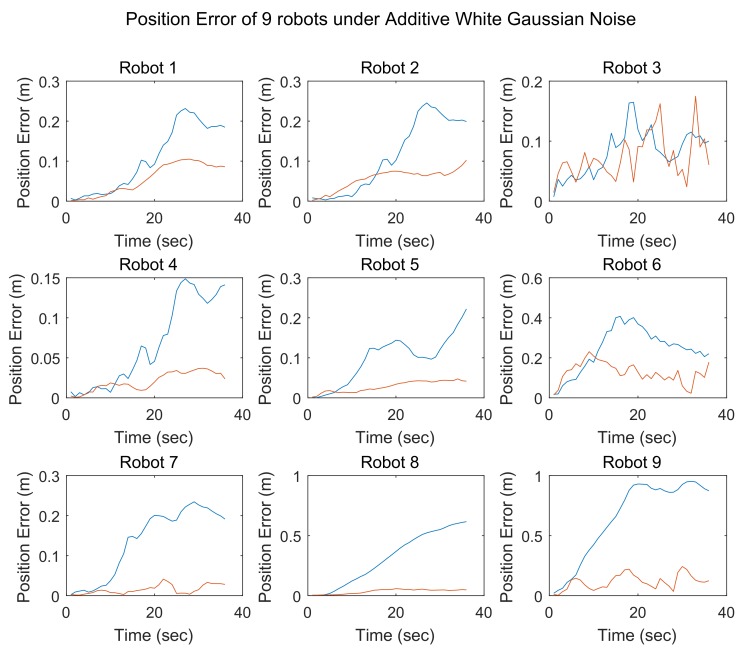
Position errors of 9 robots under additive white Gaussian noise.

The results of simulation 2 are shown in Figure 9, where each subfigure presents the localization errors for one team member as obtained using different localization methods.

The SCIFCL results will be improved if some team members achieve better positioning accuracy. However, the localization results achieved by robots with better accuracy will be “contaminated” by the robots with less position accuracy. This is mainly because the SCIFCL procedure does not consider the accuracy of the observations being fused. We divide the team members into two groups based on their localization accuracy, where the accuracy of the first group of robots is better than that of the second group. Updating the localization result for a robot in either the first or the second group with the observation from the first group of robots will improve the accuracy of its localization result. However, updating the localization result for a robot in either the first or the second group with an observation from the second group of robots will decrease the accuracy of the result being updated.

Since the SCIFCL algorithm is applied to all observations from neighboring robots, some observations from robots in the second group will be utilized to adjust the states of robots in the first group. Consequently, the localization results for robots in the first group will be “contaminated” by robots in the second group. However, with the FDI algorithm, it is possible to select the robots that belong to the second group and isolate them. If the observations from robots in the second group are excluded from the measurement update, then the accuracy achieved for the entire robot team will be enhanced.

**Figure 10:**
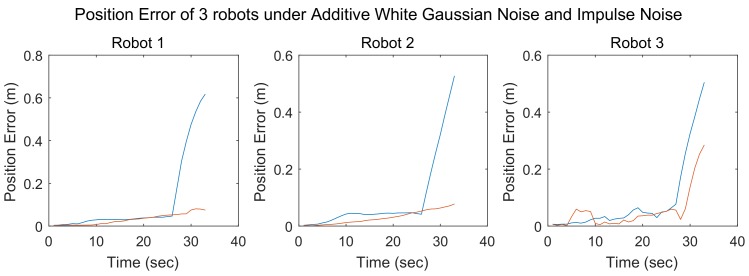
Position errors of 3 robots under additive white Gaussian noise and impulse noise.

**Figure 11:**
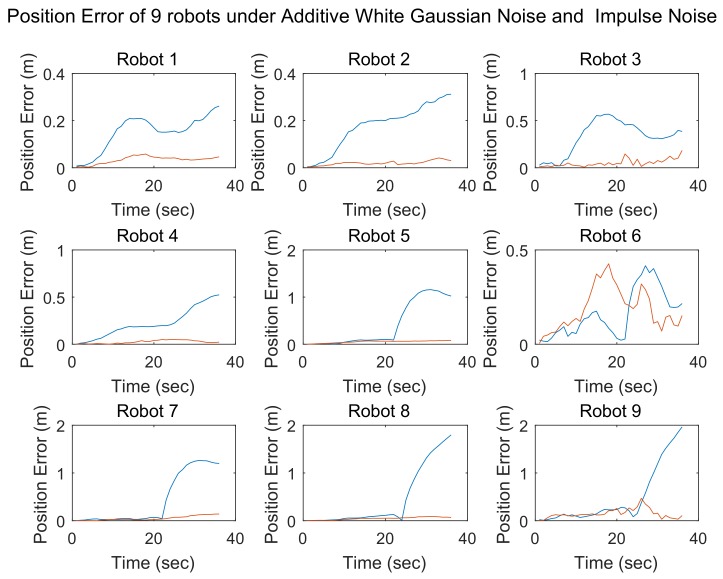
Position errors of 9 robots under additive white Gaussian noise and impulse noise.

In simulations 3 and 4, impulse noise was added in addition to additive white Gaussian noise. In simulation 3, the number of robots in the team was three, while in simulation 4, the number of team members was 9. The results of these two simulations are shown in Figure 10 and Figure 11, respectively.

As in the case of simulation 1, the localization errors achieved using different methods are represented by lines of different colors. The blue lines denote the localization errors of the SCIFCL method, and the red lines denote the localization errors of the SCIFCL method with FDI.

As shown in Figure 10 and Figure 11, the phenomenon of “contamination” is more evident when impulse noise is present. The SCIFCL results for the robot team diverge, while for the SCIFCL algorithm with FDI, the localization results for other robots in the robot team are not affected by robots that experience extreme localization failure.

The results of these four simulations demonstrate that the SCIFCL algorithm with FDI exhibits better localization performance than that of the SCIFCL algorithm alone.

## 5. Discussion

In this paper, an FDI algorithm is applied in DCL for a multirobot system. In the proposed algorithm, the signature matrix used in the original FDI algorithm is modified to enable the application of FDI in DCL, in which each robot communicates only with its neighbors. In the proposed algorithm, certain fault types in the signature matrix are combined, and a new KL residual is added. With this modified signature matrix, fault isolation can be achieved in DCL. Simulations have been conducted to test the proposed FDI algorithm for DCL. The experimental results demonstrate that the proposed DCL algorithm with FDI achieves better accuracy and robustness than the DCL algorithm without FDI does.

The purpose of the algorithm introduced in this paper is to serve as an augmentation approach to compensate for poor odometric measurements and noisy and distorted measurements from other sensors. The proposed localization method may become a valuable tool for the application of FDI in DCL.

Possible further studies are concluded below:

1. We plan to test the performance of the proposed method in a real experiment.
2. Our algorithm is designed to be an augmentation system to some localization algorithm. A localization algorithm with map for example AMCL and SCIFCL with FDI would be part of our further work.
3. In our plan, the communication graph may change but the signature matrices will not change for the algorithm FDI runs in each robot only need two nearest neighbor information. The further discussion is needed about this issue.
4. When every robot loses theirs position at the same time a re-location algorithm with sensors like GPS or anchor node will be needed.

## Figures and Tables

**Table 1:** The signature matrix of robot 1 for DCL.

	$KL^{12}$	$KL^{13}$
Odometer 1	1	1
Odometer 2	1	0
Odometer 3	0	1
Observation 1	0	0
Observation 2	1	0
Observation 3	0	1

**Table 2:** The simplified signature matrix of robot 1.

	$KL^{12}$	$KL^{13}$
Robot 1	1	1
Robot 2	1	0
Robot 3	0	1

**Table 3:** The simplified signature matrix of robot 2.

	$KL^{12}$	$KL^{23}$
Robot 1	1	0
Robot 2	1	1
Robot 3	0	1

**Table 4:** The simplified signature matrix of robot 3.

	$KL^{13}$	$KL^{23}$
Robot 1	1	0
Robot 2	0	1
Robot 3	1	1

**Table 5:** The proposed signature matrix of robot 1.

	$KL^{12}$	$KL^{13}$	$KL^{12/13}$
Robot 1	1	1	0
Robot 2	1	0	1
Robot 3	0	1	1

**Table 6:** The proposed signature matrix of robot 2.

	$KL^{12}$	$KL^{23}$	$KL^{12/23}$
Robot 1	1	0	1
Robot 2	1	1	0
Robot 3	0	1	1

**Table 7:** The proposed signature matrix of robot 3.

	$KL^{13}$	$KL^{23}$	$KL^{13/23}$
Robot 1	1	0	1
Robot 2	0	1	1
Robot 3	1	1	0
